# Prosthetic abdominal wall hernia repair in emergency surgery: from polypropylene to biological meshes

**DOI:** 10.1186/1749-7922-3-33

**Published:** 2008-12-04

**Authors:** G Campanelli, F Catena, L Ansaloni

**Affiliations:** 1 Department, of Surgery University of Insubria, Varese, Italy; 2 Department of General Surgery and Transplantation St Orsola Malpighi University Hospital, Bologna, Italy; 3 Professore Ordinario di Chirurgia dell'Università dell'Insubria di Varese, Direttore della Chirurgia Generale II, Unità Operativa di Day e Week-Surgery. Ospedale Multimedica Santa Maria di Castellanza, Viale Piemonte 70, 21053 Castellanza, Italy

## Abstract

The use of nonabsorbable prosthetic materials such as polypropylene, polyester, and ePTFE, have expanded and are now widely used in reparative surgery for abdominal wall hernias.

There are still difficulties to find correct indication for prosthetic implant in emergency hernia surgery: as a matter of fact there is still a great debate if to use non-absorbable prostheses in potentially or truly infected operating fields [e.g. after intestinal resections].

All these problems can be avoided with the use of absorbable prosthetic materials such as those composed of lactic acid polymers or lactic and glycolic acid copolymers: however, the use of these absorbable prosthesis exposes the patient to a rapid and inevitable hernia recurrence.

It is important to remember that prosthetic repair has been proven to have a significant less risk of recurrence than repair with direct sutures.

Recently, new "biologic" prosthetic materials have been developed and proposed for the clinical use in infected fields. These materials can be called "remodeling" for the way by which they are replaced after their placement within the patient. The "remodeling" process is made possible through a process of incorporation, where a reproduction of a site-specific tissue similar to the original host tissue is created.

## Commentary

In the last 30 years with the introduction of the "tension-free" techniques in hernia repair based on the use of alloplastic, nonabsorbable prosthetic materials, we have witnessed to a significant reduction in postoperative pain degree and incidence of hernia recurrences when confronted with the older nonprosthetic hernioplasties.

The use of nonabsorbable prosthetic materials such as polypropylene, polyester, and ePTFE, have hence expanded and are now widely used in reparative surgery for abdominal wall hernias [1,2]. When implanted, these nonabsorbable materials –although extremely biocompatible-stimulate a foreign-bodies reaction within the host.

After the initial inflammatory phase, the reaction is followed by an intense deposition of nonspecific fibrotic tissue and concluded by a permanent encapsulation of the alloplastic material in the host's tissues.

If these are the physiopathological bases that explain the success of alloplastic nonabsorbable prosthetic materials in hernia surgery, they are also the reasons for not uncommon complications such as infections [3-7].

Nowadays there are still difficulties to find correct indication for prosthetic implant in emergency hernia surgery: as a matter of fact there is still a great debate if to use non-absorbable prostheses in potentially or truly infected operating fields [e.g. after intestinal resections] [3].

Any area in which surgery with a possible risk of bacterial contamination is performed [bowel resections, cholecystectomy, operations on bile duct, parastomal hernias, etc], is potentially at risk for prosthetic repair. On one side there is a common consensus on what should be done in frankly contaminated areas such as in peritonitis. In fact the opinion is not to position any kind of non absorbable prosthetic material due to a very high risk of infection [do not use non absorbable materials]. On the other side it is not demonstrated that there is an increased risk of contamination of the mesh in case that simultaneous operations on the digestive tract are performed. [potentially contaminated surgical fields].

Some authors report prosthetic repair of the abdominal wall after colonic resection [potentially contaminated surgical field] with good results [1-3]. Many other perform prosthetic inguinal hernia repair in emergencies in which intestinal resection has to be made [strangulated hernias, another potentially contaminated surgical field] [4-6].

All these problems can be avoided with the use of absorbable prosthetic materials such as those composed of lactic acid polymers or lactic and glycolic acid copolymers [8].

However, the use of these absorbable prosthesis exposes the patient to a rapid and inevitable hernia recurrence as these materials, once implanted, are attacked by an inflammatory reaction that, through a hydrolytic reaction, removes and digests the implanted prosthetic material completely. In this case, the high risk of hernia recurrence is explained by the complete dissolution of the prosthetic support [8].

It is important to remember that prosthetic repair has been proven to have a significant less risk of recurrence than repair with direct sutures [9].

It also possible to perform polypropylene prosthetic incisional hernia repairs in potentially contaminated areas, with a preventive preparation of the retromuscolar-preperitoneal space where in the prosthesis implantation: then the preperitoneal space is closed temporarily suturing the peritoneum to muscular fascia after inserting iodine gauze into it. Only after preparing this space the following emergency potentially contaminating operation can be performed. Great attention must be was given not to contaminate both the peritoneal cavity and the prosthetic implant site. [3]

It is very important to underline that in incisional hernia the success of the procedure can be guaranteed only by an accurate preparation of the preperitoneal space: perfect haemostasis, temporary closure of the space inserting iodine gauzes, local antibiotic treatment, washing of the cavity and accurate drainage [3].

In general preperitoneal repair permits to have a wide vision of the inguinal, crural, and spigelian region. The dissection of this space allows to position a wide mesh that repairs the entire region with less risk of recurrence. The peritoneum also isolates the peritoneal cavity from the mesh with less risk of contamination.

A systemic antibiotic therapy should be used as routine in these cases with higher risk of infection. Many studies have proven the validity of antibiotic chemotherapy in the prevention of postoperative infections after prosthetic repair of the abdominal wall [10]. It is certain that both in non-complicated inguinal hernia and in abdominal wall hernia repairs the use of antibiotics can reduce significantly the number of infections. So particularly in operations in which we think that it is possible that enteric bacteria have contaminated the operating field we should use wide spectrum antibiotics that protect against gram + and gram – bacteria. There is no convincing evidence to suggest that the new-generation Cephalosporins are more effective than first-generation [10]. Some Authors suggest in standard prosthetic repair single dose of ampicillin and sulbactam, others Authors first – second generation cephalosporine/amoxicillin and clavulanic acid and others single dose cephtriaxone [10].

Recently, new "biologic" prosthetic materials have been developed and proposed for the clinical use in infected fields. These materials can be called "remodeling" for the way by which they are replaced after their placement within the patient. The "remodeling" process is made possible through a process of incorporation, where a reproduction of a site-specific tissue similar to the original host tissue is created. The reconstructed tissue tends to resemble the original specific tissue that replaces, not only from the histological point of view, but also functionally. Although these new prosthetic materials are all essentially composed by an extracellular matrix deprived of its cellular components and substantially differs only in relation to the source from which the extracellular matrix is obtained, whether it be either a porcine small intestine submucosa or the cadaveric human derma, or something else [9,10], they can be further subdivided in two categories: those totally remodeling that are completely substituted by a new created tissue and those partially remodeling that due to a cross-linking process don't disappear completely. The introduction of such materials in clinical practice has provided a new perspective for abdominal wall defect repair in contaminated surgical fields.

In this respect the patient can incorporate the prosthetic material by reconstructing "from himself" the specific damaged tissue and, in particular, recreate a mature "neofascia" that has a normal supportive and contenitive function. Even in the case of the abdominal wall reconstruction, the extracellular matrix implanted into the host has a direct upholding function only initially. Subsequently, it comes to be vascularized and colonized from the host cells that remodel its form until the reconstruction of a new and mature fascia is complete. Finally, this mature structure restores the original supportive and upholding function of the abdominal wall [11,12].

Although many of these products are purchased with indication for hernia repair and soft tissues reconstruction, only few of them have been reported in literature to have a clinical recognized use: among the partially remodeling the porcine dermal collagen [13-16] and among the totally remodeling the acellular extracellular matrix [17] the acellular cadaveric dermis [18-20] and the porcine small intestine submucosa [21-30].

Results of long-term studies are no longer available, especially in which they regard hernia recurrences when compared to the non-absorbable materials [some Authors reported an higher recurrent hernia incidence compared to polypropylene but these data were non confirmed].

It is already possible however, to identify clear indications to the use of this biomaterial when considering its peculiarities in the emergency hernia repair of infected or potentially infected fields or in patients with high risk of infection of the non-absorbable prosthesis [i.e. immune-depressed subject] [31-50].

## Competing interests

The authors declare that they have no competing interests.

## Authors' contributions

GC participated in the design of the paper, FC conceived the paper, and participated in its design and coordination., LA conceived the paper, and participated in its design and coordination. All authors read and approved the final manuscript.
